# Mfn2 is responsible for inhibition of the RIG-I/IRF7 pathway and activation of NLRP3 inflammasome in Seneca Valley virus-infected PK-15 cells to promote viral replication

**DOI:** 10.3389/fimmu.2022.955671

**Published:** 2022-07-25

**Authors:** HuiDan Deng, Song Zhu, Ling Zhu, Jing Sun, YuChun Ding, FengQin Li, ZhiJie Jian, Jun Zhao, LiShuang Deng, JunLiang Deng, YouTian Deng, HongRui Guo, XianGang Sun, Si Yuan Lai, HuaQiao Tang, HengMin Cui, Liang Peng Ge, ZhiWen Xu

**Affiliations:** ^1^ College of Veterinary Medicine, Sichuan Agricultural University, Chengdu, China; ^2^ Key Laboratory of Animal Disease and Human Health of Sichuan Province, Sichuan Agricultural University, Chengdu, China; ^3^ National Center of Technology Innovation for Pigs, Chongqing, China; ^4^ College of Animal Science, Xichang University, Xichang, China

**Keywords:** SVV, Mfn2, NLRP3 inflammasome, RIG-I signaling pathway, innate immune response

## Abstract

Seneca Valley virus (SVV), a non-enveloped positive single-stranded virus can cause vesicular disease in swine. However, the mechanisms by which SVV activates an innate immune response remain unknown. Mitofusin-2 (MFN2), a mitochondria-shaping protein regulating mitochondrial fusion and fission, plays a crucial role in innate immune responses. But, the roles of Mfn2 in SVV infection have not been elucidated. Here, we show that SVV inhibited Mfn2 expression and NLRP3 inflammasome, activating RIG-I/IRF7 signaling pathway to increase IFN-λ3 expression. Overexpression of Mfn2 inhibited RIG-I/IRF7 signaling pathway, thus decreasing IFN-λ3 expression and promoting SVV replication. Interestingly, overexpression of Mfn2 also activated NLRP3 inflammasome but did not inhibit SVV proliferation. That may mean the RIG-I/IRF7 signaling pathway plays a more important role in SVV proliferation in PK-15 cells. This study could provide important insights into the modulation of host metabolism during SVV infection and provide a strong theoretical basis for a better understanding of the pathogenic mechanism and immune activation mechanism of SVV.

## Introduction

Seneca Valley virus (SVV) is a positive single-stranded RNA virus belonging to the picornavirus family. The virus was discovered by accident in 2002 in a culture of adenovirus type 5 vectors in PER cell lines. C6, named Seneca Valley Virus 001 (SVV-001) (1). It is speculated that the agent may be introduced into cell culture through fetal bovine serum or porcine trypsin. The latter is considered more likely because abundant viruses serologically associated with SVV-001 have been isolated from pigs in the United States over the past 20 years (2). Studies on SVV-001 focused on its oncolytic activity in tumor therapy at first. In recent years, SVV infection in swine has been reported in the United States, Canada, Brazil, China and other countries (3–5). SVV infection can increase the mortality of newborn piglets and cause fluid-filled/ruptured vesicles and ulcerative lesions at the snout, coronary band, and hooves, as well as anorexia and lameness in adult pigs (6, 7). In 2015, the first Chinese SVV strain was isolated from pigs in Guangdong Province. Since then, more and more cases of SVV infection have been reported in other provinces of China and led to serious economic losses, indicating that SVV has spread rapidly and widely in China (8, 9).

An innate immune response such as retinoic acid-inducible gene I (RIG-I) like receptors (RLRs) signaling and inflammasomes is the first line of host defense in response to pathogens. According to the traditional paradigm, after the virus gets across the mucus, the virus can be sensed by the pattern recognition receptors (PRRs), triggering the production of interferons (IFNs) which induce the expression of hundreds of IFN-stimulated genes (ISGs) that block viral replication and further virus spread (10). Some PRRs, such as NACHT, LRR, PYD domains-containing protein 1 (NLRP1), NLRP3, NLR family CARD domain-containing protein 4 (NLRC4) and absent in melanoma 2 (AIM2), recruit apoptosis-associated speck-like proteins (ASC) and caspase-1 to form inflammasomes to initiate inflammation and some forms of cell death. Thus, participation in antiviral response (11). Numerous studies indicate that SVV disrupts the host defense system in virus-infected cells. Qian et al. (12) and Wen et al. (13) found that in human embryonic kidney 293T cells. SVV 2C and 3C protein induces cleavage of MAVS, TRIF, TANK and degradation of RIG-I, blocks activation of the RLR pathway and inhibits the production of type I interferon, meanwhile, SVV infection can induce host cell apoptosis to promote virus replication. In PK-15 cells, SVV 3Cpro reduces IRF3 and IRF7 protein expression to block the transcription of interferons (IFNs) such as IFN-β, IFN-α1, IFN-α4 and ISG54 to escape the host’s intrinsic innate immune system (14). In macrophages and pigs, SVV 3D binds with NLRP3 to activate the NLRP3 inflammasome, on the other hand, SVV 3D protein interacts with IKKα and IKKβ to induce NF-κB activation. Thus promoting IL-1 β transcription and secretion (15). Our previous studies have proved that SVV can activate innate immune response *via* RIG-I signaling pathway (16). However, the details on how SVV activates innate immunity are still not clear.

Mitofusin2 (Mfn2), a mitochondrial outer membrane that participates in the initial step of mitochondrial fusion and promotes the maintenance of cellular homeostasis (17, 18), regulates various other biological processes such as cell proliferation and cell death (19–21). Numerous studies have shown that Mfn2 is a key regulator of innate immune responses during viral infections. Earlier study reported that Mfn2 inhibited antiviral immune responses in hepatitis B virus related hepatocellular carcinoma (22). However, in HIV-1 Vpr infected HEK293 cells, Mfn2 overexpression can alleviating cell death *via* mediating ER-Mitochondria Interaction (23). Nonetheless the role of Mfn2 in SVV infecting PK-15 cells has not been studied yet.

Therefore, our studies were carried out to investigate the possible mechanisms of how Mfn2 affects immune response in PK-15 cells infection with SVV and find out the relationship between Mfn2, inflammasome and RIG-I signaling pathway. Our study provides a foundation and a new insight for future systematic exploring of SVV infection mechanism.

## Materials and methods

### Virus and cells

PK-15 cells (kept at Key Laboratory of Animal Disease and Human Health of Sichuan Province, Sichuan Agricultural University, Chengdu) were maintained in Dulbecco’s Modified Eagle’s Medium Nutrient Mixture (DMEM) (Gibco, USA), supplemented with antibiotics (100 units/ml penicillin and 100µg/ml streptomycin), and 10% fetal bovine serum (FBS) (Gibco, USA). The SVV was maintained at Key Laboratory of Animal Disease and Human Health of Sichuan Province, Sichuan Agricultural University, Chengdu. The virus was propagated in PK-15 cell with 2% FBS (Gibco, USA) added in the DMEM.

### Transfection

The Mfn2 was cloned into vector pcDNA3.1 (kept at Key Laboratory of Animal Disease and Human Health of Sichuan Province, Sichuan Agricultural University, Chengdu). For transfection, the cells were transiently transfected with vector pcDNA3.1 or plasmids encoding Mfn2 using Lipofectamine 3000 (Invitrogen, 2185325) following the manufacturer’s protocols. Cell lysates were collected after 24 h to verify overexpression efficiency by western blot.

### Western blot assay

After PK-15 cells were lysed, total proteins were extracted with RIPA buffer (Thermo Fisher Scientific) and Thermo Scientific Halt protease inhibitor cocktail. Protein concentration was measured by BCA protein assay kit (Thermo Fisher Scientific). Equal amounts of protein sample were loaded into 12% SDS-PAGE and transferred to nitrocellulose filter membranes. Then, the membrane was blocked in nonfat dry milk (5%) for 1 h at RT. Membranes were incubated with the primary antibodies overnight at 4°C followed by one hour of incubation using proper secondary HRP-conjugated antibodies (Bio-Rad) and development with ECL detection kit (GE Healthcare, Piscataway, NJ, USA). Then, the membranes were detected with Bio-Rad ChemiDoc XRS+System (Bio-Rad Laboratories, Inc., Hercules, CA, USA). The primary antibodies were RIG-I polyclonal antibody (CST, United States), anti-Phospho-IRF7 (Bioss, China), GAPDH (Abcam, United States), Rabbit Anti-ASC antibody (Proteintech, United States), cleaved Rabbit Anti-IL-1β antibody (CST, United States), Rabbit Anti-IL-18 antibody (CST, United States), Rabbit Caspase 1/p20/p10 Polyclonal antibody (proteintech, United States); Mfn2 polyclonal antibody (proteintech, United States) and NLRP3 polyclonal antibody (proteintech, United States). The second antibody was anti-Rabbit or mouse IgG-HRP (Sangon, China). At least three biological replicates were analyzed for each experiment.

### RNA extraction and quantitative real-time PCR

RNAiso Plus (9109; Takara, China) was used for extracting the PK-15 cells total RNA following protocols provided by the manufacturer. RNA (1μg) was used to synthesize cDNA through Prim-ScriptTM RT reagent Kit (RR047A, Takara, China) following specific instructions. Primers were designed and synthesized by Sangon (Shanghai, China). The mRNA expression was measured with SYBR^®^ Premix Ex TaqTMII (RR820A, Takara, China). The reactions protocol was 95°C for 10 min, followed by under 95°C for 10 min, under 60°C for 20 s, and under 72°C for 20 s. The qRT-PCR data were analyzed using the 2-ΔΔCT method (21). At least three biological replicates were analyzed for each experiment. The qRT-PCR primers were shown in **Table 1**.

**Table 1:** Primer sequences of genes selected for analysis SVV replication.

Target gene	Primer	Primer Sequence (5’->3’)	Tm(°C)
SVV-VP1	Forward Reverse	AGGTACTGGAGAAGGACGCT GGTTGACGTACAGGCCGAAA	57

### ELISA assay

After treated with SVV for 24 h, the culture supernate was collected for IFN-λ1 and IFN-λ3 detection. Test was carried out according to the kit brochures operation (Enzyme Industrial Co., Ltd, China) and the spectrophotometric absorbance was assessed at 450 nm for IFN-λ1 and IFN-λ3.

### Immunocytochemistry

Immunocytochemistry was performed to detect SVV proliferation *in vitro*. PK-15 cells were cultured in 24-well plates and treated with SVV for 24 h. After that, cells were washed with PBS and fixed with 4% PFA at RT for 10 min. Then, cells were incubated with 0.1% Triton X-100 at RT for 10 min followed by blocking with 1% BSA at RT for 1 h. Next, cells were incubated with rabbit anti-VP1 antibody (saved in our lab) overnight at 4 °C. After washing with PBS, cells were incubated with secondary antibodies rabbit IgG (Thermo Scientific) at RT for 1 h. Cells were analyzed using fluorescence microscopy (PhotoFluor LM-75, 89 North, Burlington, VT, USA). At least three biological replicates for each experiment were performed and representative images are shown.

### Statistical analysis

Data are expressed as mean ± standard deviation. All statistical analyses were analyzed by GraphPad Prism (GraphPad Software, Inc). Unpaired T-test and One-way analysis of variance (ANOVA) were used to investigate the significance of differences between the experimental groups and the control group. p < 0.05 indicates statistical significance of the difference. Each experiment was repeated at least three times.

## Results

### SVV decreased Mfn2 expression and inhibited NLRP3 inflammasome activation

**Figure 1:**
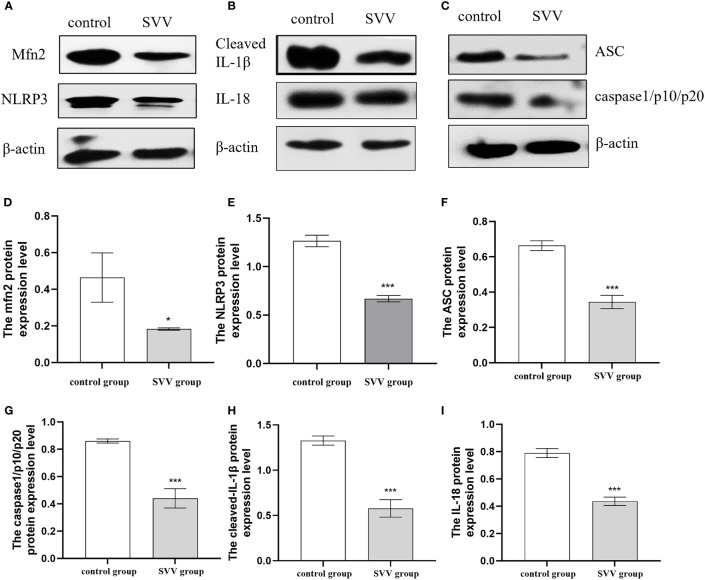
SVV decreased Mfn2 expression, inhibited NLRP3 inflammasome activation. **(A–C)** The western blot assay for Mfn2, NLRP3, ASC, caspase1/p10/p20, cleaved-IL-1β and IL-18. **(D–I)** The relative protein expression level of Mfn2, NLRP3, ASC, caspase1/p10/p20, cleaved-IL-1β and IL-18. Unpaired T-test were used to investigate the significance of differences between the experimental groups and the control group *; P < 0.05, ***; P < 0.005,.

PK-15 cells were infected with SVV strain SVV-SC-01 at the multiplicity of infection (MOI) of 2 for 24 h. As shown in **Figure 1**, SVV decreased Mfn2 protein expression level (P < 0.05) in comparison with control group. At the same time, the NLRP3, ASC, caspase1/p10/p20, cleaved-IL-1β and IL-18 protein expression also decreased (P < 0.005) in SVV infection group when compared to control group. That means the NLRP3 inflammasome activation was inhibited by SVV infection.

### The inhibition of RIG-I/IRF7 signaling pathway promote SVV proliferation in PK-15 cells

**Figure 2:**
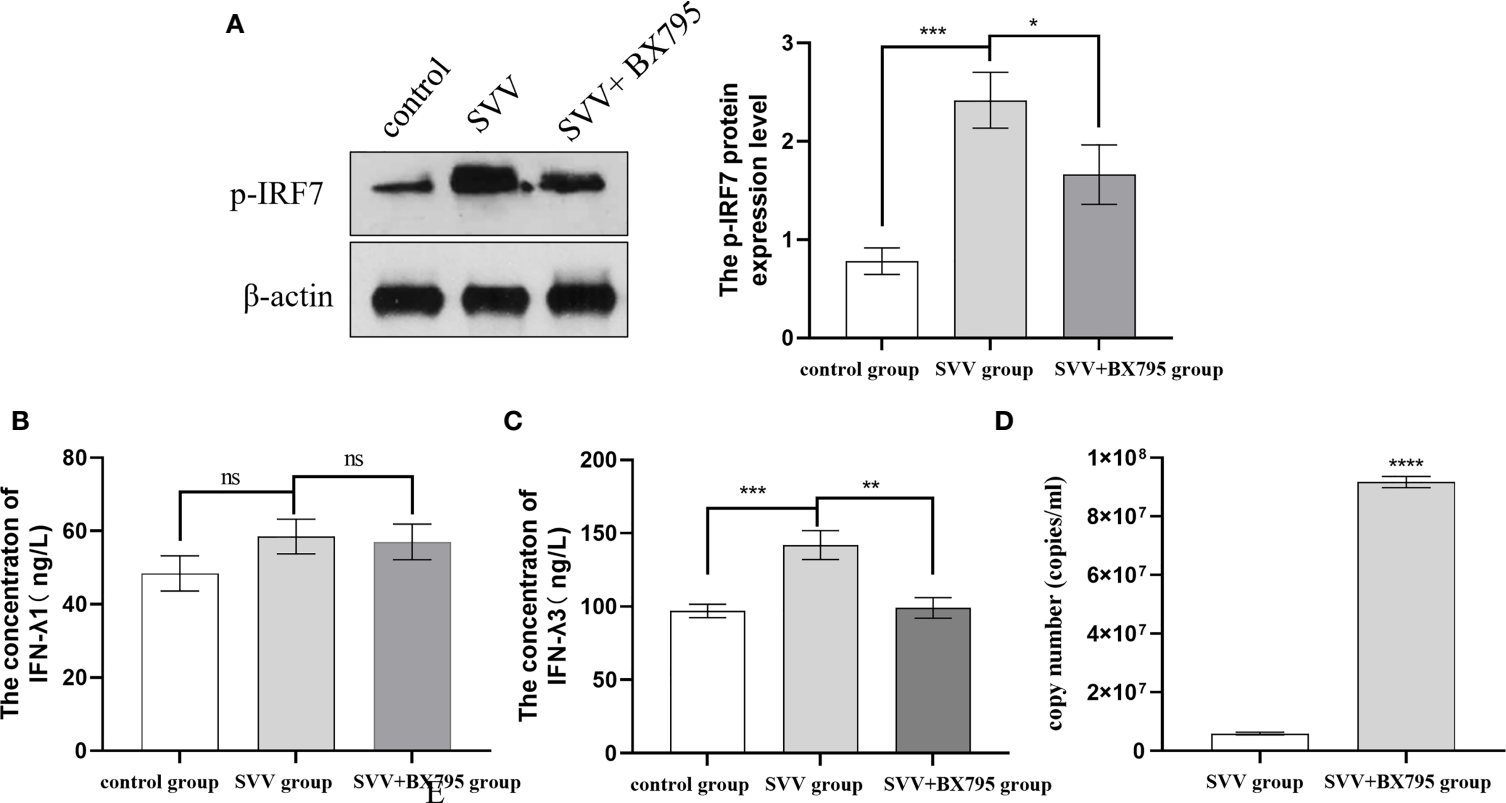
IRF7 inhibitor (BX795) inhibited IRF7 phosphorytion and IFN-λ3 secretion thus promote SVV proliferation. **(A)** The western blot assay for p-IRF7. **(B)**The relative protein expression level of IRF3. **(C, D)** The Elisa assay for IFN-λ1 and IFN-λ3. **(E)** The qRT-PCR assay for SVV copy number. One-way analysis of variance (ANOVA) were used to investigate the significance of differences between the SVV groups and the SVV+BX795 group or between the control group and SVV group *; P < 0.05, **; P < 0.01, ***; P < 0.005, ****; P < 0.001, ns means no significance.

Our previous results also showed that SVV infection activated RIG-I/IRF7 signaling pathway shown as increased RIG-I and p-IRF7 protein expression level, 0.5 μmol/L IRF7 inhibitor (BX795) decreased IFN-λ1 and IFN-λ3 mRNA expression levels (16). But how the content of IFN-λ1 and IFN-λ3 in the supernatant changed after SVV and BX795 treated still unknown. To further investigated the role of RIG-I/IRF7 signaling in IFN-λs seretion and SVV proliferation. We use 0.5 μmol/L IRF7 inhibitor to treated cells 1 h before treated with SVV. The results showed that SVV only increased IFN-λ3 content (*P < 0.005*). After inhibited IRF7 phosphorylation, only IFN-λ3 secretion was inhibited (*P < 0.01*). The qRT-PCR results showed SVV proliferation was enhanced (*P < 0.001*) after BX795 treatment in PK-15 cells. That means the IFN-λ3 may be the key factor in RIG-I/IRF7 mediated SVV proliferation. The results were shown in **Figure 2**.

### The activation of NLRP3 inflammasome inhibited SVV proliferation in PK-15 cells

**Figure 3:**
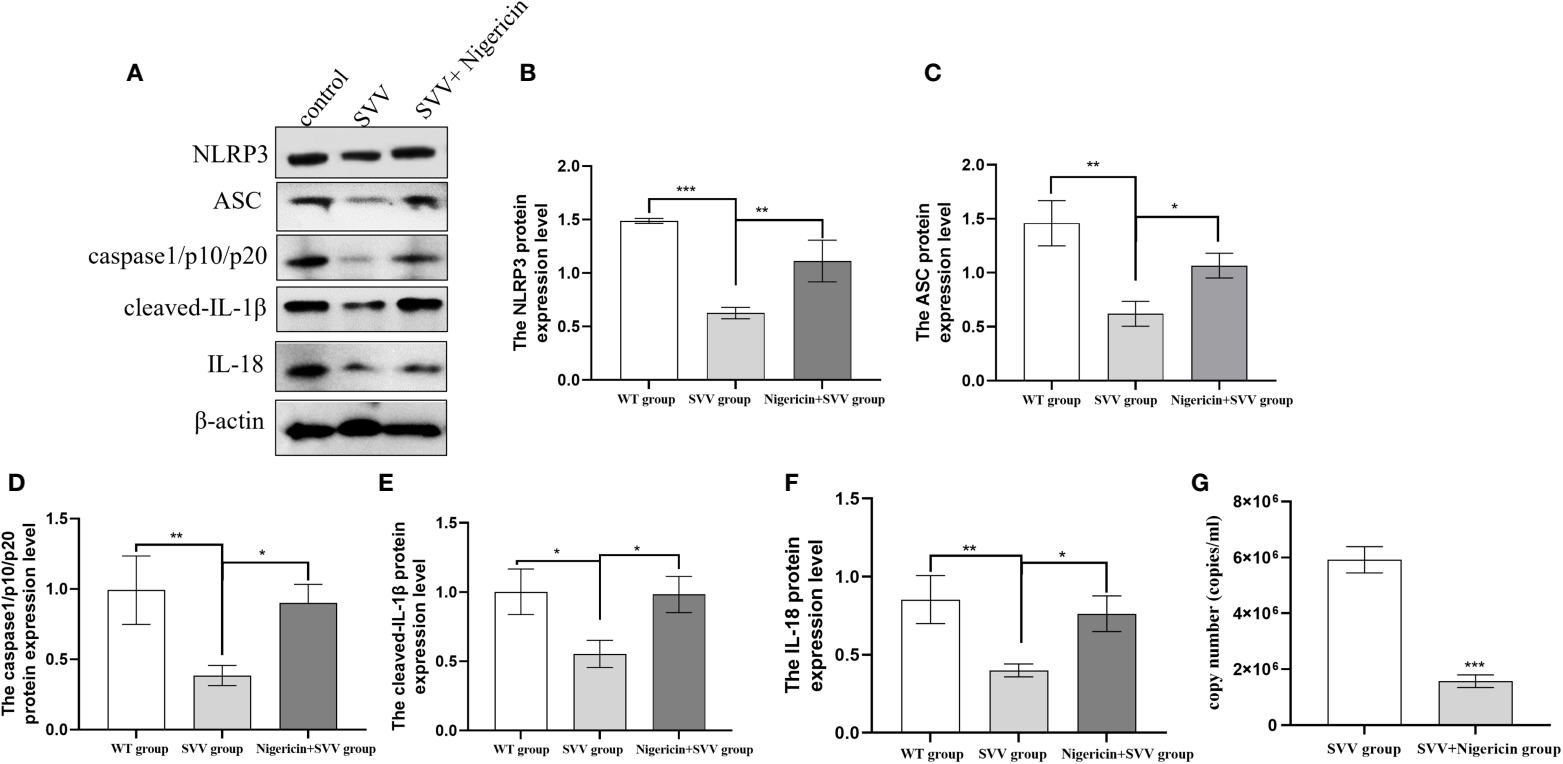
NLRP3 inflammasome activator (Nigeration) relieved SVV inhibited NLRP3 inflammasome activation and suppress SVV proliferation in PK-15 cells **(A)** The western blot assay for NLRP3, ASC, caspase1/p10/p20, cleaved-IL-1β and IL-18. **(B–F)**The relative protein expression level of NLRP3, ASC, caspase1/p10/p20, cleaved-IL-1β and IL-18. **(G)** The qRT-PCR assay for SVV copy number. One-way analysis of variance (ANOVA) were used to investigate the significance of differences between the SVV groups and the SVV+BX795 group or between the control group and SVV group *; P < 0.05, **; P < 0.01, ***; P < 0.005.

In order to further understand the role of NLRP3 inflammasome in SVV proliferation. We use NLRP3 inflammasome activator Nigeration to treated cells with SVV for 24 h. As shown in **Figure 3**. 10 μmol/L Nigeration relieved SVV inhibited NLRP3 inflammasome activation. The NLRP3, ASC, caspase1/p10/p20, cleaved-IL-1β and IL-18 protein expression level all increased (*P < 0.01 or P < 0.05*) when compared with SVV infection group. In addition, the qRT-PCR results showed that 10 μmol/L Nigeration inhibited the SVV proliferation in PK-15 cells. These results demonstrated that activation of NLRP3 inflammasome protect PK-15 cells from SVV infection.

### The Mfn2 overexpression enhanced SVV proliferation and promoted cell damage in PK-15 cells

**Figure 4:**
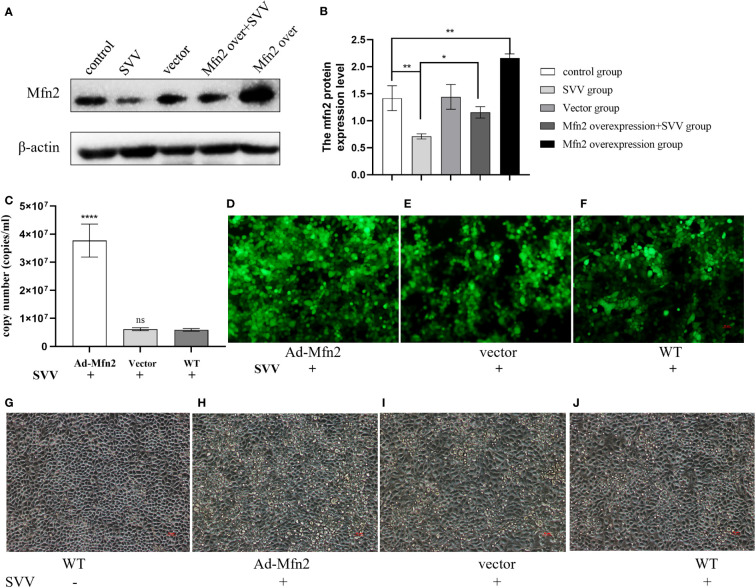
Mfn2 overexpression accelerate SVV proliferation and promote cell damage in PK-15 cells **(A)** The western blot assay for Mfn2. **(B)** The relative protein expression level of Mfn2. **(C)** The qRT-PCR assay for SVV copy number. **(D–F)** The immunocytochemistry assay for SVV detection. **(G–J)** The change of PK-15 cells morphology after Mfn2 overexpression. One-way analysis of variance (ANOVA) were used to investigate the significance of differences between the WT and other groups. *; P < 0.05, **; P < 0.01, ****; P < 0.001, ns means no significance.

To explore the function of Mfn2 in SVV infection in BHK-21. The Mfn2 was cloned into vector pcDNA3.1. The the cells were transiently transfected with vector pcDNA3.1 (vector group) or plasmids encoding Mfn2 (Ad-Mfn2 group) using Lipofectamine 3000 for 24 h. Then, cells were infected with SVV for 24 h. **Figures 1A, B** showed that Mfn2 protein was successfully transfected into PK-15 cells. qRT-PCR and Immunocytochemistry results showed that Mfn2 overexpression increased SVV proliferation (*P < 0.001*). And the cellular damage was increased in Ad-Mfn2 group when compared with WT group under microscopy (**Figure 4**).

### The Mfn2 overexpression inhibit RIG-I/IRF7 signaling pathway and decreased IFN-λ3 content

**Figure 5:**
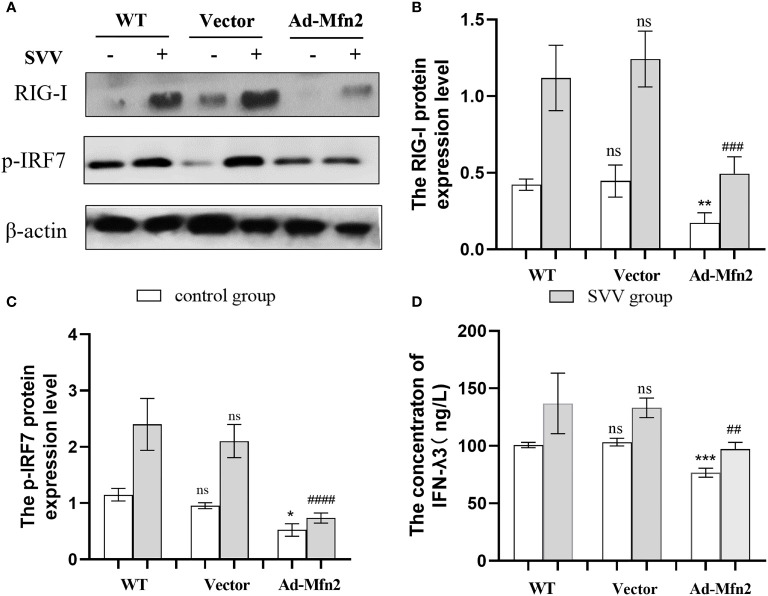
Mfn2 overexpression restrain SVV-induced RIG-I/IRF7 protein expression level and IFN-λ3 content in supernatant in PK-15 cells **(A)** The western blot assay for RIG-I and p-IRF7. **(B, C)** The relative protein expression level of RIG-I and p-IRF7. **(D)** The Elisa assay for IFN-λ3 detection. Unpaired T-test and One-way analysis of variance (ANOVA) were used to investigate the significance of differences. * means the difference between the WT and Ad-Mfn2 in control group; # means the difference between the WT and Ad-Mfn2 in SVV group. *; P < 0.05, **^,##^; P < 0.01, ***^,###^; P < 0.005, ^####^; P < 0.001, ns means no significance.

As shown in **Figure 5**, Mfn2 overexpression (Ad-Mfn2) decreased (*P < 0.001 or P < 0.005*) the RIG-I and p-IRF7 protein expression when compared with WT group after SVV infection. And the Elisa assay results also showed that the IFN-λ3 content in the supernatant in Ad-Mfn2 group was also decreased (*P < 0.01*) compared to WT group after SVV infection.

### The Mfn2 overexpression activated NLRP3 inflammasome

**Figure 6:**
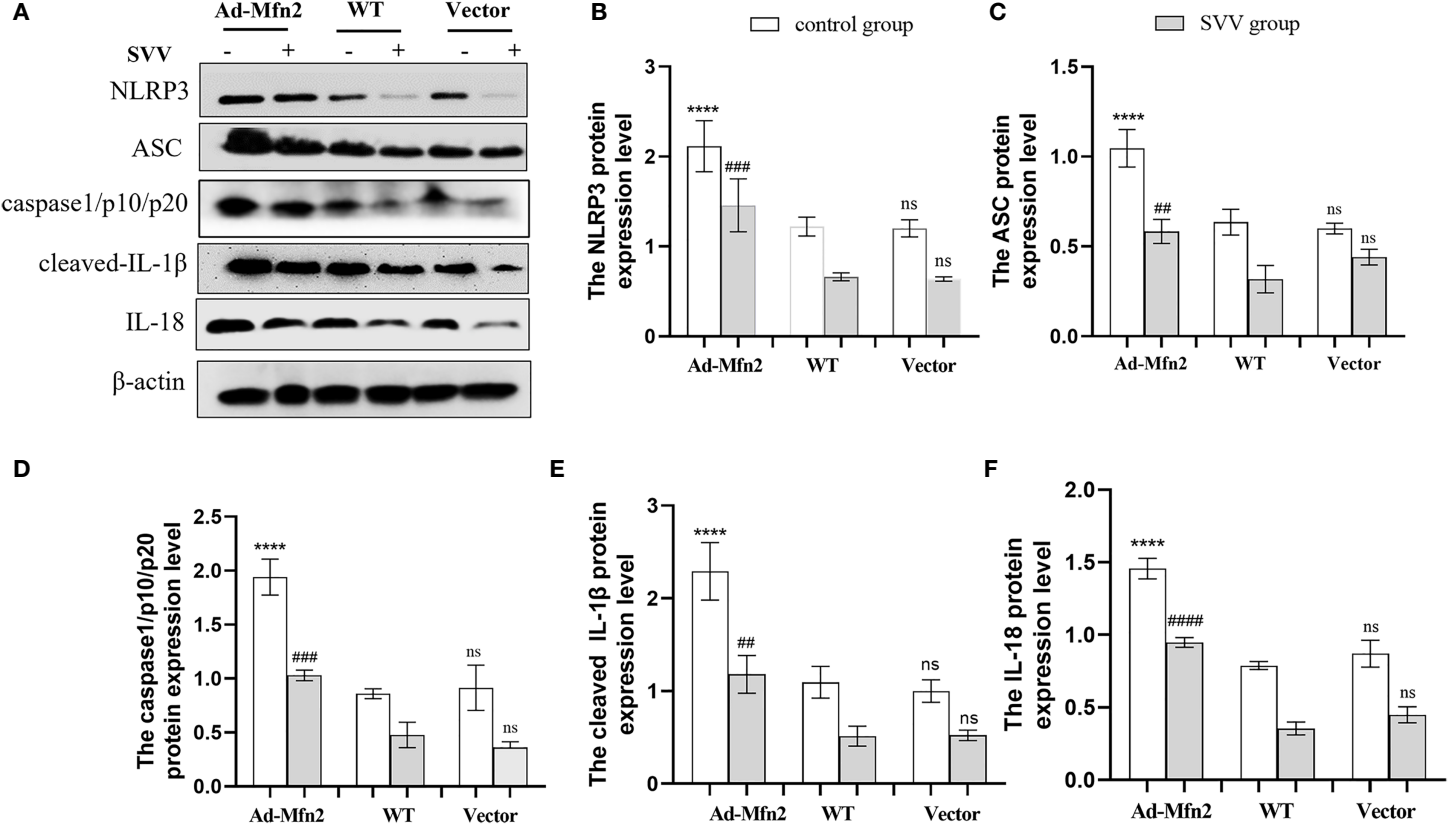
Mfn2 overexpression induced SVV-inhibited NLRP3 inflammasome activation in PK-15 cells. **(A)** The western blot assay for NLRP3, ASC, caspase1/p10/p20, cleaved-IL-1β and IL-18. **(B–F)** The relative protein expression level of NLRP3, ASC, caspase1/p10/p20, cleaved-IL-1β and IL-18. One-way analysis of variance (ANOVA) were used to investigate the significance of differences. * means the difference between the WT and Ad-Mfn2 in control group; # means the difference between the WT and Ad-Mfn2 in SVV group; and other groups. ^##^; P < 0.01, ^###^; P < 0.005, ****^,####^; P < 0.001, ns means no significance.

As illustrated in **Figure 6**, after SVV infection in PK-15 cells, the NLRP3 inflammasome signaling pathway was up-regulated in Ad-Mfn2 group compared to WT group. Concretely reflected in the increased protein expression levels of NLRP3, ASC, caspase1/p10/p20, cleaved-IL-1β and IL-18 in Ad-Mfn2 group when compare with WT group during SVV infection (*P < 0.0001 or P < 0.005 or P < 0.01*).

## Discussion

Seneca Valley virus (SVV) is a newly type of virus in pig industry in China, the first outbreak of SVV occurred in Guangdong Province in March 2015 and then spread into other province (24). SVV can cause vesicular disease and epidemic transient neonatal death in swine. The typical clinical symptoms include vesicular, ulcerative lesions on the snout, oral mucosa, coronary bands and hooves (25). Since the outbreak of SVV in China, Numerous studies have focused on innate immune response. The results proved that SVV inhibits the production of type I interferon through a variety of pathways. The SVV infection does not trigger the host’s early innate immune response and the production of type I interferon in human embryonic kidney 293T cells, and its 3C protein induces the cleavage of receptor molecules of type I interferon pathways MAVS, TRIF and TANK through protease activity, blocking the activation of RLR pathway and inhibiting the production of type I interferon (12). Meanwhile, SVV 3C protease inhibits the expression of RIG-1, TBK1 and TRAF3 by degrading IRF3 and IRF7 or acting as a de-ubiquitination enzyme, and inhibits the type I interferon pathway (14), thus evading the innate immunity of the host against virus. The host protein RIG-I is responsible for activating type I interferon pathway to inhibit viral replication in SVV-infected porcine cells (26). Our previous study demonstrated that SVV induced RIG-I/IRF7 signaling pathway activation. In this study, we found that SVV inhibits Mfn2 protein expression and NLRP3 inflammasome activation. To further explore the role of Mfn2, NLRP3 inflammasome and RIG-I in SVV infection in PK-15 cells, we have conducted a series of studies shown as below.

The innate immune response mediated by RNA virus involves the RLRs signaling pathway (27). The RNA virus can be recognized by RIG-I, and then activates downstream related signal pathways to exert innate antiviral immunity such as phosphorylates IRF3 and IRF7. Phosphorylated IRF3 and IRF7 form homologous and/or heterologous dimers that are transported to the nucleus and bind to IFN-stimulated response elements (ISREs) to induce IFNs and ISGs (28–30). In HEK-293T, SW620 and SK6 cells, SVV inhibit type I interferon production by degrading RIG-I (12, 13) and IRF-7 (14). Li Pengfei (26) has proved that knock out RIG-I in PK-15 cells reduced type I interferon production. Our previous study was first carried out to investigate the relationship between RIG-I/IRF7 pathway and type III interferon. We found that inhibition of IRF7 inhibitor inhibits IFN-λ1 and IFN-λ3 mRNA expression in PK-15 cells (16). In this study, we found that inhibition of IRF7 phosphorylation promote SVV replication but only reduced IFN-λ3 content in supernatant. These results illustrated that RIG-I/IRF7 meidated IFN-λ3 production not IFN-λ1 play an important role in anti-viral immunity.

The NLRP3 is one of the member of PRRs activate innate immune system in response to harmful stimuli (31–33). NLRP3 inflammasome is essential for host immune defense against viral infections (34). The NLRP3 interacts with the ASC to initiate inflammasome assembly. Promotes pro-caspase-1 recruitment to the inflammasome complex and activates caspase-1. Activated caspase-1 cleaves the cytokines interleukin-1 β (pro-IL-1β) and IL-18 into mature and biologically active forms. Thus, promoting immune response. Wen wei (25) demonstrated that in SK6 cells, SVV could induce pyroptosis. However, in late infection, SVV may reduced caspase-1 expression because 3C^pro^ cleave NLRP3. In pig bone marrow-derived macrophages (BMDMs), SVV infection activate NLRP3 to induces IL-1β secretion and production (15). On the contrary, our data illustrated that SVV infection inhibits the activation of NLRP3 inflammasome shown as down-regulation of NLRP3, ASC, caspase-1/p10/p20, cleaved-IL-1β and IL-18. The difference of the results between our study and others may because of the different cells and the different in the origin of viral strain. To further investigate the role of NLRP3 inflammasome in SVV replication. We use activator of NLRP3 inflammasome Nigericin to treat cells with SVV. The results showed after NLRP3 inflammasome activtation, the SVV proliferation was inhibited. Declared that NLRP3 inflammasome play an anti-viral function in SVV infection PK-15 cells.

Mfn2 is a master regulator of immune responses during viral infections (35). Studies have proved that on one hand, Mfn2 inhibited antiviral immune responses by interacting with MAVs or mediating RLR signaling and IRF3 expression during encephalomyocarditis virus (EMCV), Measles, VSV, H1N1 infection (36, 37). During infection with human immunodeficiency type 1 (HIV-1) in macrophages, Mfn2 was up-regulated by TREM1. And the TREM1-dependent MFN2 upregulation contribute to the ability of HIV-1 survival in host cells (38). Interestingly, Mfn2 also have anti-viral functions. After dengue virus infection, Mfn2 are cleaved by dengue virus protease NS2B3, and the Mfn2 keeps MMP to inhibit cell death from dengue virus infection (39). In addition, Mfn2 binds to NLRP3 to promote IL-1β secretion after infection with RNA viruses, including influenza, measles, or EMCV (40). In this study, we found that SVV infection decreased Mfn2 protein expression. Overexpression of Mfn2 inhibited RIG-I/IRF7 signaling pathway and restrain IFN-λ3 secretion, thus promoting SVV replication. Mfn2 overexpression also activate NLRP3 inflammasome. Based on aboved mentioned results, activation of NLRP3 inflammasome inhibited SVV replication. However, Mfn2 overexpression promote SVV proliferation. These results may demonstrate that RIG-I/IRF7 signaling pathway is more important than NLRP3 inflammasome in anti-SVV response. And the reason may because IFN-λ plays the most important role in mucosal antiviral immune response (41, 42). After all, IFN-λ is produced earlier and more frequently, has strong antiviral activity and does not mediate inflammation. Therefore, many side effects are avoided (43).

## Conclusion

**Figure 7:**
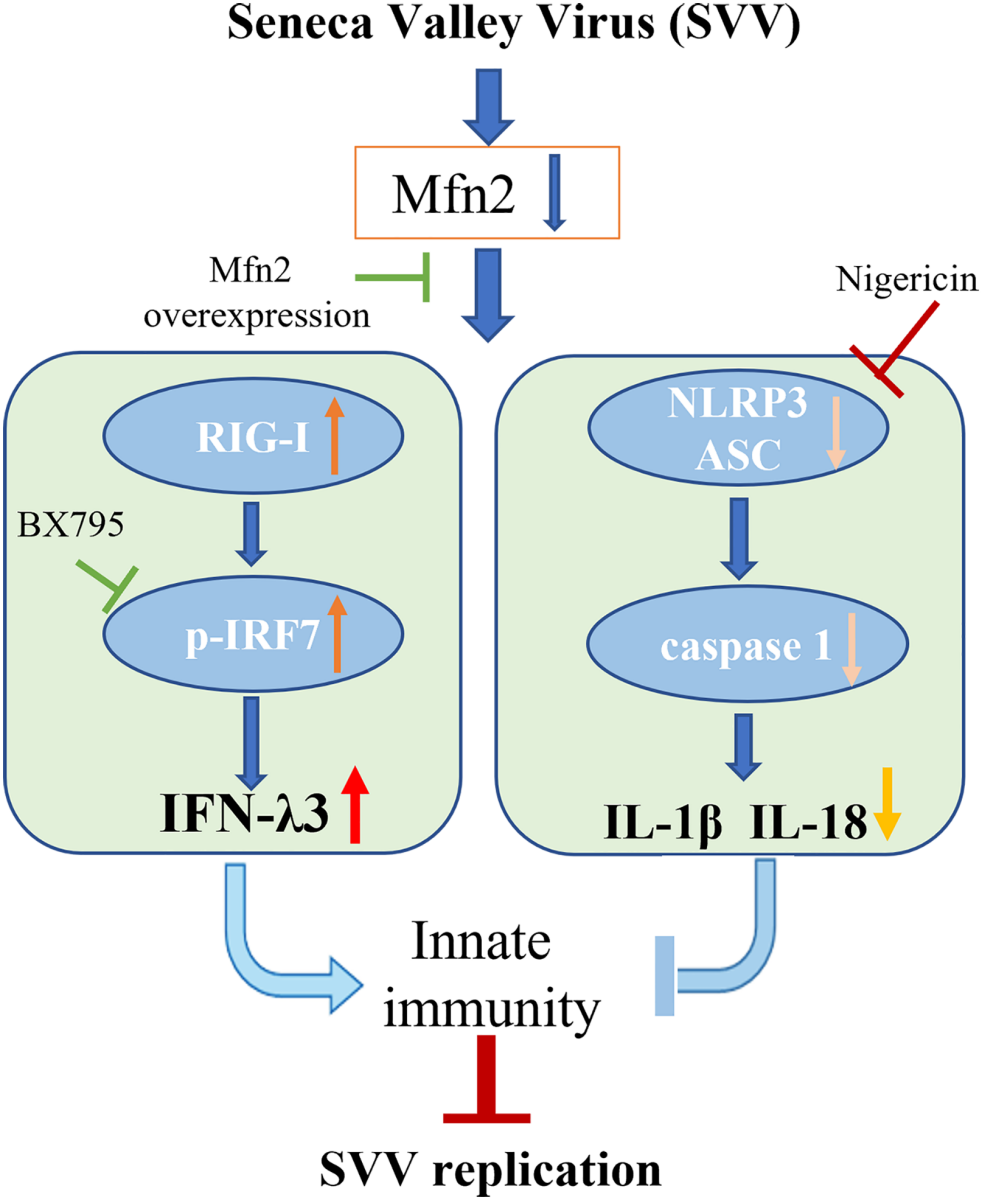
Schematic diagram of the possible mechanism of Mfn2 antiviral response. Mfn2 promote SVV proliferation mostly *via* down-regulating RIG-I/IRF7-dependent IFN-λ3 protein expression levels.

Our study demonstrated that Mfn2 inhibited antiviral activity against SVV infection in PK-15 cells. Mfn2 promote SVV proliferation *via* down-regulating RIG-I/IRF7-dependent IFN-λ3 protein expression levels. Also, the activation of NLRP3 inflammasome alone play an antiviral function in SVV infection. However, Mfn2-dependent NLRP3 inflammasome did not inhibit SVV replication (**Figure 7**). That may be because of the inhibition of IFN-λ3 secretion during Mfn2 overexpression. Our findings suggest that deficiency of Mfn2 may represent a promising therapeutic target for SVV prevention in the future, and the IFN-λ3 may be the most important therapeutic agent for SVV prevention and treatment.

## Data availability statement

The original contributions presented in the study are included in the article/supplementary material. Further inquiries can be directed to the corresponding author/s.

## Author contributions

HD, LZ and ZX conceived and designed the experiments. HS, SZ, ZJ performed the experiments. LD, FL, XS, SL and JZ performed the analysis. HD drafted the manuscript. ZX, JD, YDeng, HT, HC and HG substantively revised this manuscript. JS, YDing and LG helped to revised manuscript according to reviewer comments and polish the article. All authors read and approved the final manuscript.

## Funding

This work was supported by the Science and Technology Department of Sichuan Province, China [grant number 2021ZDZX0010] and [grant number 2020YFN0147], Science and Technology Department of Chongqing, China [grant number cstc2021jscx-dxwtBX0007]. The funders had no role in study design, data collection and analysis, decision to publish, or preparation of the manuscript.

## Acknowledgments

We thank Prof. Zhu for her helpful discussions.

## Conflict of interest

The research was conducted in the absence of any commercial or financial relationships that could be construed as a potential conflict of interest.

## Publisher’s note

All claims expressed in this article are solely those of the authors and do not necessarily represent those of their affiliated organizations, or those of the publisher, the editors and the reviewers. Any product that may be evaluated in this article, or claim that may be made by its manufacturer, is not guaranteed or endorsed by the publisher.

## References

[1] Hales LM Knowles NJ Reddy PS Xu L Hay C Hallenbeck PL. Complete genome sequence analysis of seneca valley virus-001, a novel oncolytic picornavirus. J Gen Virol (2008) 89(Pt 5):1265–75. doi: 10.1099/vir.0.835700 18420805

[2] Segalés J Barcellos D Alfieri A Burrough E Marthaler D. Senecavirus a. Vet Pathol (2017) 54(1):11–21. doi: 10.1177/0300985816653990 27371541

[3] Canning P Canon A Bates JL Gerardy K Linhares DC Piñeyro PE. Neonatal mortality, vesicular lesions and lameness associated with senecavirus a in a U.S. sow farm. Transbound Emerg Dis (2016) 63(4):373–8. doi: 10.1111/tbed.12516 PMC7169707 27213868

[4] Pasma T Davidson S Shaw SL. Idiopathic vesicular disease in swine in Manitoba. Can Vet J = La Rev Vet Can (2008) 49(1):84–5. PMC2147704 18320985

[5] Vannucci FA Linhares DC Barcellos DE Lam HC Collins J Marthaler D. Identification and complete genome of seneca valley virus in vesicular fluid and sera of pigs affected with idiopathic vesicular disease, Brazil. Transbound Emerg Dis (2015) 62(6):589–93. doi: 10.1111/tbed.12410 26347296

[6] Baker KL Mowrer C Canon A Linhares DC Rademacher C Karriker LA. Systematic epidemiological investigations of cases of senecavirus a in us swine breeding herds. Transbound Emerg Dis (2017) 64(1):11–8. doi: 10.1111/tbed.12598 27888583

[7] Leme RA Oliveira TE Alcântara BK Headley SA Alfieri AF Yang M. Clinical manifestations of senecavirus a infection in neonatal pigs, Brazil, 2015. Emerg Infect Dis (2016) 22(7):1238–41. doi: 10.3201/eid2207.151583 PMC4918167 27315157

[8] Qian S Fan W Qian P Chen H Li X. Isolation and full-genome sequencing of seneca valley virus in piglets from China, 2016. Virol J (2016) 13(1):173. doi: 10.1186/s12985-016-0631-2 27756396 PMC5069920

[9] Zhu Z Yang F Chen P Liu H Cao W Zhang K. Emergence of novel seneca valley virus strains in China, 2017. Transbound Emerg Dis (2017) 64(4):1024–9. doi: 10.1111/tbed.12662 28544501

[10] Sun Y Jiang J Tien P Liu W Li J. Ifn-Λ: a new spotlight in innate immunity against influenza virus infection. Protein Cell (2018) 9(10):832–7. doi: 10.1007/s13238-017-0503-6 PMC6160391 29332267

[11] Man SM Kanneganti TD. Converging roles of caspases in inflammasome activation, cell death and innate immunity. Nat Rev Immunol (2016) 16(1):7–21. doi: 10.1038/nri.2015.7 26655628 PMC4915362

[12] Qian S Fan W Liu T Wu M Zhang H Cui X. Seneca Valley virus suppresses host type i interferon production by targeting adaptor proteins mavs, trif, and tank for cleavage. J Virol (2017) 91(16):e00823–17. doi: 10.1128/jvi.00823-17 PMC5533933 28566380

[13] Wen W Yin M Zhang H Liu T Chen H Qian P. Seneca Valley virus 2c and 3c inhibit type i interferon production by inducing the degradation of rig-i. Virology (2019) 535:122–9. doi: 10.1016/j.virol.2019.06.017 31299488

[14] Xue Q Liu H Zhu Z Yang F Ma L Cai X. Seneca Valley virus 3c(pro) abrogates the irf3- and irf7-mediated innate immune response by degrading irf3 and irf7. Virology (2018) 518:1–7. doi: 10.1016/j.virol.2018.01.028 29427864

[15] Choudhury SM Ma X Zeng Z Luo Z Li Y Nian X. Senecavirus a 3d interacts with nlrp3 to induce il-1β production by activating nf-κb and ion channel signals. Microbiol Spectr (2022) 10(2):e0209721. doi: 10.1128/spectrum.02097-21 35254168 PMC9045273

[16] Peng K Deng L Wei J Zhao J Deng H Tao Q. Transcriptome analyses of senecavirus a-infected pk-15 cells: rig-i and irf7 are the important factors in inducing type iii interferons. Front Microbiol (2022) 13:846343. doi: 10.3389/fmicb.2022.846343 35308346 PMC8931416

[17] Eura Y Ishihara N Yokota S Mihara K. Two mitofusin proteins, mammalian homologues of fzo, with distinct functions are both required for mitochondrial fusion. J Biochem (2003) 134(3):333–44. doi: 10.1093/jb/mvg150 14561718

[18] Filadi R Pendin D Pizzo P. Mitofusin 2: from functions to disease. Cell Death Dis (2018) 9(3):330. doi: 10.1038/s41419-017-0023-6 29491355 PMC5832425

[19] Xin Y Li J Wu W Liu X. Mitofusin-2: a new mediator of pathological cell proliferation. Front Cell Dev Biol (2021) 9:647631. doi: 10.3389/fcell.2021.647631 33869201 PMC8049505

[20] Wang W Cheng X Lu J Wei J Fu G Zhu F. Mitofusin-2 is a novel direct target of p53. Biochem Biophys Res Commun (2010) 400(4):587–92. doi: 10.1016/j.bbrc.2010.08.108 20804729

[21] Zhang GE Jin HL Lin XK Chen C Liu XS Zhang Q. Anti-tumor effects of mfn2 in gastric cancer. Int J Mol Sci (2013) 14(7):13005–21. doi: 10.3390/ijms140713005 PMC3742171 23797661

[22] Wang X Liu Y Sun J Gong W Sun P Kong X. Mitofusin-2 acts as biomarker for predicting poor prognosis in hepatitis b virus related hepatocellular carcinoma. Infect Agents Cancer (2018) 13:36. doi: 10.1186/s13027-018-0212-7 PMC6258311 30498519

[23] Huang CY Chiang SF Lin TY Chiou SH Chow KC. Hiv-1 vpr triggers mitochondrial destruction by impairing mfn2-mediated er-mitochondria interaction. PloS One (2012) 7(3):e33657. doi: 10.1371/journal.pone.0033657 22438978 PMC3306277

[24] Liu F Wang Q Huang Y Wang N Shan H. A 5-year review of senecavirus a in china since its emergence in 2015. Front Vet Sci (2020) 7:567792. doi: 10.3389/fvets.2020.567792 33134352 PMC7561413

[25] Wen W Li X Wang H Zhao Q Yin M Liu W. Seneca Valley virus 3c protease induces pyroptosis by directly cleaving porcine gasdermin d. J Immunol (Baltimore Md 1950) (2021) 207(1):189–99. doi: 10.4049/jimmunol.2001030 34183365

[26] Li P Zhang X Cao W Yang F Du X Shi Z. Rig-I is responsible for activation of type i interferon pathway in seneca valley virus-infected porcine cells to suppress viral replication. Virol J (2018) 15(1):162. doi: 10.1186/s12985-018-1080-x 30352599 PMC6199795

[27] Eisenächer K Krug A. Regulation of rlr-mediated innate immune signaling–it is all about keeping the balance. Eur J Cell Biol (2012) 91(1):36–47. doi: 10.1016/j.ejcb.2011.01.011 21481967

[28] Saha SK Pietras EM He JQ Kang JR Liu SY Oganesyan G. Regulation of antiviral responses by a direct and specific interaction between traf3 and cardif. EMBO J (2006) 25(14):3257–63. doi: 10.1038/sj.emboj.7601220 PMC1523175 16858409

[29] Honda K Taniguchi T. Irfs: master regulators of signalling by toll-like receptors and cytosolic pattern-recognition receptors. Nat Rev Immunol (2006) 6(9):644–58. doi: 10.1038/nri1900 16932750

[30] Chang MX. The negative regulation of retinoic acid-inducible gene i (rig-i)-like receptors (rlrs) signaling pathway in fish. Dev Comp Immunol (2021) 119:104038. doi: 10.1016/j.dci.2021.104038 33548290

[31] Takeuchi O Akira S. Pattern recognition receptors and inflammation. Cell (2010) 140(6):805–20. doi: 10.1016/j.cell.2010.01.022 20303872

[32] Sharma D Kanneganti TD. The cell biology of inflammasomes: mechanisms of inflammasome activation and regulation. J Cell Biol (2016) 213(6):617–29. doi: 10.1083/jcb.201602089 PMC4915194 27325789

[33] Lamkanfi M Dixit VM. Mechanisms and functions of inflammasomes. Cell (2014) 157(5):1013–22. doi: 10.1016/j.cell.2014.04.007 24855941

[34] Allen IC Scull MA Moore CB Holl EK McElvania-TeKippe E Taxman DJ. The nlrp3 inflammasome mediates *in vivo* innate immunity to influenza a virus through recognition of viral rna. Immunity (2009) 30(4):556–65. doi: 10.1016/j.immuni.2009.02.005 PMC2803103 19362020

[35] Tur J Pereira-Lopes S Vico T Marín EA Muñoz JP Hernández-Alvarez M. Mitofusin 2 in macrophages links mitochondrial ros production, cytokine release, phagocytosis, autophagy, and bactericidal activity. Cell Rep (2020) 32(8):108079. doi: 10.1016/j.celrep.2020.108079 32846136

[36] Yasukawa K Oshiumi H Takeda M Ishihara N Yanagi Y Seya T. Mitofusin 2 inhibits mitochondrial antiviral signaling. Sci Signaling (2009) 2(84):ra47. doi: 10.1126/scisignal.2000287 19690333

[37] Luo Z Liu LF Jiang YN Tang LP Li W Ouyang SH. Novel insights into stress-induced susceptibility to influenza: corticosterone impacts interferon-β responses by mfn2-mediated ubiquitin degradation of mavs. Signal Transduct Targeted Ther (2020) 5(1):202. doi: 10.1038/s41392-020-00238-z PMC7499204 32943610

[38] Campbell GR To RK Spector SA. Trem-1 protects hiv-1-infected macrophages from apoptosis through maintenance of mitochondrial function. mBio (2019) 10(6):e02638–19. doi: 10.1128/mBio.02638-19 PMC6851287 31719184

[39] Yu CY Liang JJ Li JK Lee YL Chang BL Su CI. Dengue virus impairs mitochondrial fusion by cleaving mitofusins. PloS Pathog (2015) 11(12):e1005350. doi: 10.1371/journal.ppat.1005350 26717518 PMC4696832

[40] Ichinohe T Yamazaki T Koshiba T Yanagi Y. Mitochondrial protein mitofusin 2 is required for Nlrp3 inflammasome activation after rna virus infection. Proc Natl Acad Sci USA (2013) 110(44):17963–8. doi: 10.1073/pnas.1312571110 PMC3816452 24127597

[41] Mordstein M Neugebauer E Ditt V Jessen B Rieger T Falcone V. Lambda interferon renders epithelial cells of the respiratory and gastrointestinal tracts resistant to viral infections. J Virol (2010) 84(11):5670–7. doi: 10.1128/jvi.00272-10 PMC2876583 20335250

[42] Pott J Mahlakõiv T Mordstein M Duerr CU Michiels T Stockinger S. Ifn-lambda determines the intestinal epithelial antiviral host defense. Proc Natl Acad Sci USA (2011) 108(19):7944–9. doi: 10.1073/pnas.1100552108 PMC3093475 21518880

[43] Phillips S Mistry S Riva A Cooksley H Hadzhiolova-Lebeau T Plavova S. Peg-interferon lambda treatment induces robust innate and adaptive immunity in chronic hepatitis b patients. Front Immunol (2017) 8:621. doi: 10.3389/fimmu.2017.00621 28611778 PMC5446997

